# An implementation of spin–orbit coupling for band structure calculations with Gaussian basis sets: Two-dimensional topological crystals of Sb and Bi

**DOI:** 10.3762/bjnano.9.94

**Published:** 2018-03-28

**Authors:** Sahar Pakdel, Mahdi Pourfath, J J Palacios

**Affiliations:** 1 School of Electrical and Computer Engineering, University College of Engineering, University of Tehran, Tehran 14395-515, Iran; 2 Departamento de Física de la Materia Condensada, Universidad Autónoma de Madrid, 28049 Madrid, Spain; 3 School of Nano Science, Institute for Research in Fundamental Sciences (IPM), Tehran 19395-5531, Iran; 4 Institute for Microelectronics, TU Wien, Gusshausstrasse 27–29/E360, 1040 Vienna, Austria; 5 Instituto Nicolás Cabrera (INC), and Condensed Matter Physics Institute (IFIMAC), Universidad Autónoma de Madrid, 28049 Madrid, Spain.

**Keywords:** antimonene, electronic structure, Sb few-layers, spin–orbit coupling (SOC), topological material

## Abstract

We present an implementation of spin–orbit coupling (SOC) for density functional theory band structure calculations that makes use of Gaussian basis sets. It is based on the explicit evaluation of SOC matrix elements, both the radial and angular parts. For all-electron basis sets, where the full nodal structure is present in the basis elements, the results are in good agreement with well-established implementations such as VASP. For more practical pseudopotential basis sets, which lack nodal structure, an ad-hoc increase of the effective nuclear potential helps to capture all relevant band structure variations induced by SOC. In this work, the non-relativistic or scalar-relativistic Kohn–Sham Hamiltonian is obtained from the CRYSTAL code and the SOC term is added a posteriori. As an example, we apply this method to the Bi(111) monolayer, a paradigmatic 2D topological insulator, and to mono- and multilayer Sb(111) (also known as antimonene), the former being a trivial semiconductor and the latter a topological semimetal featuring topologically protected surface states.

## Introduction

The topological character of topological materials (mostly insulators but also non-insulators) in most relevant cases originates from relativistic corrections that cannot be neglected in the Hamiltonian of heavy elements, more specifically from spin–orbit coupling (SOC). Such materials are usually characterized by non-zero topological invariants that can be either computed simply from the parity of the Bloch wave functions in centrosymmetric crystals or from other more involved implementations in non-centrosymmetric systems [1–6]. Topological materials typically feature a band inversion. In a gedanken experiment, one can imagine tuning the SOC at will. As the SOC is increased from zero towards its nominal value, it pushes up the valence band while bringing down the conduction band of the imaginary SOC-free material. In this process, the gap closes and reopens again, giving rise to the non-zero topological invariant.

The essential features of the band structure of topological materials (at least the elemental ones) can be obtained from the tight-binding (TB) model where the Hamiltonian is built through a Slater–Koster [7] atomic parametrization. These models, however, are usually restricted to the description of valence electrons, implicitly by assuming a minimal basis set of spd orbitals. The SOC is included by adding the matrix elements of the 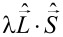 operator where λ is taken as an atomic parameter [8]. Although the simplicity of TB modeling is appealing, this method is obviously restricted to a limited set of problems. TB parameters are available for most elemental materials [9], but not in general for all compound materials (which is the case of most topological insulators). The versatility of this model is also limited by the sensitivity of the TB parameters to the specific structural variations which also needs to be parametrized [10].

On the opposite side of sophistication, the electronic structure of topological materials can be evaluated through density functional theory (DFT). According to the type of basis sets, DFT codes fall into two broad categories: those making use of plane-waves and those using localized orbitals. Arguably, the most reliable implementations of SOC can be found in the code FLEUR [11] and also in codes such as Vienna Ab initio Simulation Package [12] (VASP) or QuantumEspresso [13–14] (QE), all of them employing plane-waves for the interstitial or valence electrons, while approaching the core electrons differently. Since localized orbitals are convenient for a number of reasons, for instance for quantum transport calculations [15–16], a Kohn–Sham Hamiltonian obtained from plane-wave DFT codes may be transformed into a TB-like Hamiltonian by changing to a basis of Wannier functions [17–18]. While the results of this transformation can be accurate, they are not straightforward to carry out. On the other hand, self-consistent implementations of SOC for codes using localized orbitals for valence electrons are, however, much less common [19–20].

In most currently available implementations, including those using localized orbitals basis sets, the SOC is effectively introduced through pseudopotentials [19–20]. Here, we propose a different route, employing the actual shape of the basis functions. In particular we present an implementation of SOC for DFT calculations based on Gaussian-type localized basis sets, attempting to bridge the gap between the simplicity of TB Hamiltonians with their one-parameter implementation of SOC and the accuracy and transferability of a DFT-level description of the band structure. We make use of the non-relativistic (or scalar relativistic) Kohn–Sham Hamiltonian, here obtained using the CRYSTAL code [21–23], to which we add the SOC a posteriori. The matrix elements are explicitly evaluated for both radial and angular parts of the basis elements, by using the screened nuclear potential. For the radial part, we rely on the actual analytical expressions of the Gaussian-type basis elements, as employed in codes such as CRYSTAL, Gaussian [24], Nwchem [25], etc. Among the available basis sets, all-electron (AE) basis sets [26], featuring the full nodal structure of the orbitals and able to properly capture SOC effects, might not be well designed for band structure calculation of solids in general or appear inefficient due to their computational cost. Here we show that when AE basis sets work properly at the band structure level in calculations without SOC, accurate results can be obtained from our proposed implementation. Alternatively, basis sets using effective core potentials or pseudopotentials, which reproduce better band structures and are computationally less demanding, lack nodal structure near the nucleus. This has prompted us to modify the nuclear potential through a fitting multiplicative factor to effectively model the SOC effect. Importantly, despite the fact that we are dealing with different types of orbitals of different shells, only a single parameter is needed since the relative values of the matrix elements are properly captured.

As possibly relevant examples, we have chosen to apply our implementation to Sb and Bi, which are prototypical topological materials where SOC plays a crucial role. Despite being elemental, they present a broad range of behaviors. While bulk Bi is a trivial semimetal, a Bi(111) monolayer is a 2D topological insulator (TI) [27]. Sb few-layers in the (111) direction, typically for more than ≈7 layers, behave as a 3D topological semimetal, while the Sb(111) monolayer is a trivial indirect-gap semiconductor. In order for our SOC implementation to be of practical use, it should capture these trivial/non-trivial topological transitions and give the most faithful representation of the electronic band structure for any number of layers. This includes the presence of helical and topologically protected edge or surface states. For comparison, and as a reliable reference, we make use of the band structures obtained from the well-established plane-wave code VASP. In general, we find a very satisfactory agreement between the band structures calculated by our approach for both AE (without parameters), pseudopotential (single parameter) basis sets, and the VASP results, proving ours to be a practical a posteriori implementation of SOC once a standard non-relativistic or scalar relativistic DFT calculation based on localized orbitals has been performed.

## Methodology

### Gaussian basis sets

The accuracy of electronic structure calculations is limited, not only by functional, but also by the basis set used to expand the wave functions. When working with localized basis sets, it is crucial to choose a large enough number of elements or a set of properly chosen ones. Typically, the basis functions are centered on atoms, and are so called ”atomic orbitals”. Two types of atomic orbital functions are typically employed in molecular orbital calculations, namely, Slater type orbitals (STOs) and Gaussian type orbitals (GTOs). Slater [7] introduced STOs as basis functions due to their similarity with the eigenfunctions of the hydrogen atom. They possess an exponential decay at long range and Kato’s cusp [28] condition at short range. Their general definition is

[1]
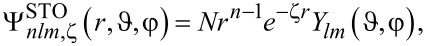


where *N* is the normalization constant. The radial part is characterized by the principal quantum number *n* and the exponent ζ while the angular part is given by the spherical harmonics which are orthogonal to the radial part and characterized by *l* and *m*, the azimuthal and magnetic quantum numbers, respectively. The ζ parameter, is variationally optimized with respect to the total energy of each atom. STOs have the advantage of a direct physical interpretation and are thus naturally good basis for molecular orbitals. However, from a computational point of view, STOs are not competitive. In practice, the radial part of STOs is approximated by a linear combination of GTOs (or primitives). Spherical GTOs were proposed by Boys [29] with a radial part defined as

[2]
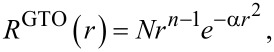


where the exponent α determines the extension of the function. Huzinaga [30] has illustrated that it is adequate to consider *n* = *l* + 1 and hence optimized GTO basis sets use 1s functions to represent all s-type orbitals, 2p functions for p-type, etc. Despite the computational benefits, GTOs have two major disadvantages, namely, they do not have a cusp at the nucleus and they fall off to zero too rapidly for large radius. However, these shortcomings can be overcome by considering linear combinations of GTOs to form contracted Gaussian-type orbitals (CGTOs):

[3]
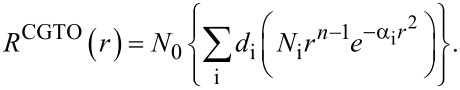


Here each primitive, as defined in Equation 2, is normalized on its own (*N*_i_) and the whole contracted function has an overall normalization constant (*N*_0_). The coefficients *d*_i_ and exponents α_i_ determine the radial shape of the CGTO. A large enough number of primitives with coefficients *d*_i_ of different signs can reproduce the expected atomic nodal behavior of wave functions near the nucleus. Introducing the nodal structure in the basis sets turns out to be irrelevant for most band structure calculations and increases the computational effort, significantly. However, as we will show in the next section, for the calculation of SOC, the exact behavior of the wave functions near the core is required.

### Evaluation of SOC matrix elements

The output Hamiltonian and overlap matrices of the CRYSTAL code, ignoring broken spin-symmetry solutions, are the same for up and down spin electrons. SOC is considered to be a purely intra-atomic interaction. Rigorous approximations to the full relativistic Dirac–Kohn–Sham Hamiltonian, which decouple the electronic part from the positronic part, yield to lowest order a SOC correction of the form 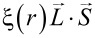 (see, e.g., [31] for a nice overview of a fairly extensive topic) which mixes orbital angular momentum (*m*) and spin (σ) quantum numbers. Since the angular and radial parts of the wave functions are orthogonal, SOC matrix elements between different CGTOs can be straightforwardly evaluated as

[4]
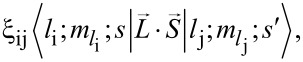


where 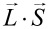 acts on the spin degree of freedom and the spherical harmonics, while the radial contribution can be obtained from

[5]
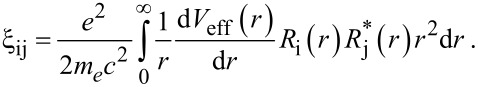


Here *R*_i_(*r*) is the radial part of the i-th atomic CGTO (built as described in the previous section) and *V*_eff_(*r*) is the effective screened nuclear potential that electrons actually feel. Here we are not concerned with the rigorous discussion concerning the approximations that lead to Equation 5 and the origin of *V*_eff_ (for details see [31]). It suffices to say that, intuitively, the potential must be of the form *Z*/*r* very close to the core and behave as 1/*r* far apart. For the case of an isolated atom, it has been shown that making use of the unscreened nuclear potential will result in an over estimation of SOC splittings. A simple model has also been suggested for screened nuclear potential, which includes the screening by adding an orbital dependent charge term (placed at the origin) to the bare nuclear potential [32]. The effective potential can also be extracted from an atomic DFT calculation. Here, we explored both possibilities and found no significant differences.

A correct electronic band structure in solids requires an accurate description of chemical bondings and hence, enough variational flexibility in the valence region. On the other hand, since the main contribution to the SOC matrix elements stems from the vicinity of the nucleus, a correct description of orbitals is also essential near the core. AE basis sets specifically designed for the latter purpose are common in atomic physics and molecular chemistry. While they can capture the full nodal structure of the orbitals, it is, however, unclear how well they perform when it comes to the band structure of solids, which is our main concern here. Our results indicate that, when AE basis sets band structures are in good agreement with those of plane-wave calculations before including SOC (which might not be always the case), fairly accurate results can be obtained after including SOC. We have also found out that a proper renormalization of the effective potential makes even pseudopotential basis sets (without nodal structure) suitable for band structure calculations where SOC plays an important role.

## Results and Discussion: Elemental topological Materials, Sb and Bi 2D Crystals

### Antimonene

Antimonene, a term generically used for Sb(111) in 2D form, has been recently added to the growing library of 2D crystals. Its recent isolation and characterization [33], is bringing this material into the focus of the research community. Several DFT studies on this material have predicted a number of exciting physico-chemical properties, including a tunable band gap with potential applications in optoelectronics [34–37], low thermal conductance with low electrical resistivity for energy generation through thermoelectricity [38], and exotic topological features under strain [39–41]. However, it was not until last year that few experimental works brought all those expectations closer to reality [33]. It was demonstrated that it is possible to isolate few or even single stable layers of antimonene, in ambient conditions. Moreover, new procedures such as liquid exfoliation and epitaxial growth methods were reported.

Theoretical works on antimonene can be divided into two categories. The most recent publications refer to monolayer antimonene (or occasionally bilayer antimonene) and can be found in the context of new 2D crystals. Other works, which go a few years back in time, refer to few-layered (FL) antimonene (or Sb(111) thin films), and can be found in the context of 3D TIs [1]. The physical properties of antimonene evolve quite drastically from mono- to few-layer cases, and each deserves a separate discussion.

#### Monolayer antimonene

**Figure 1:**
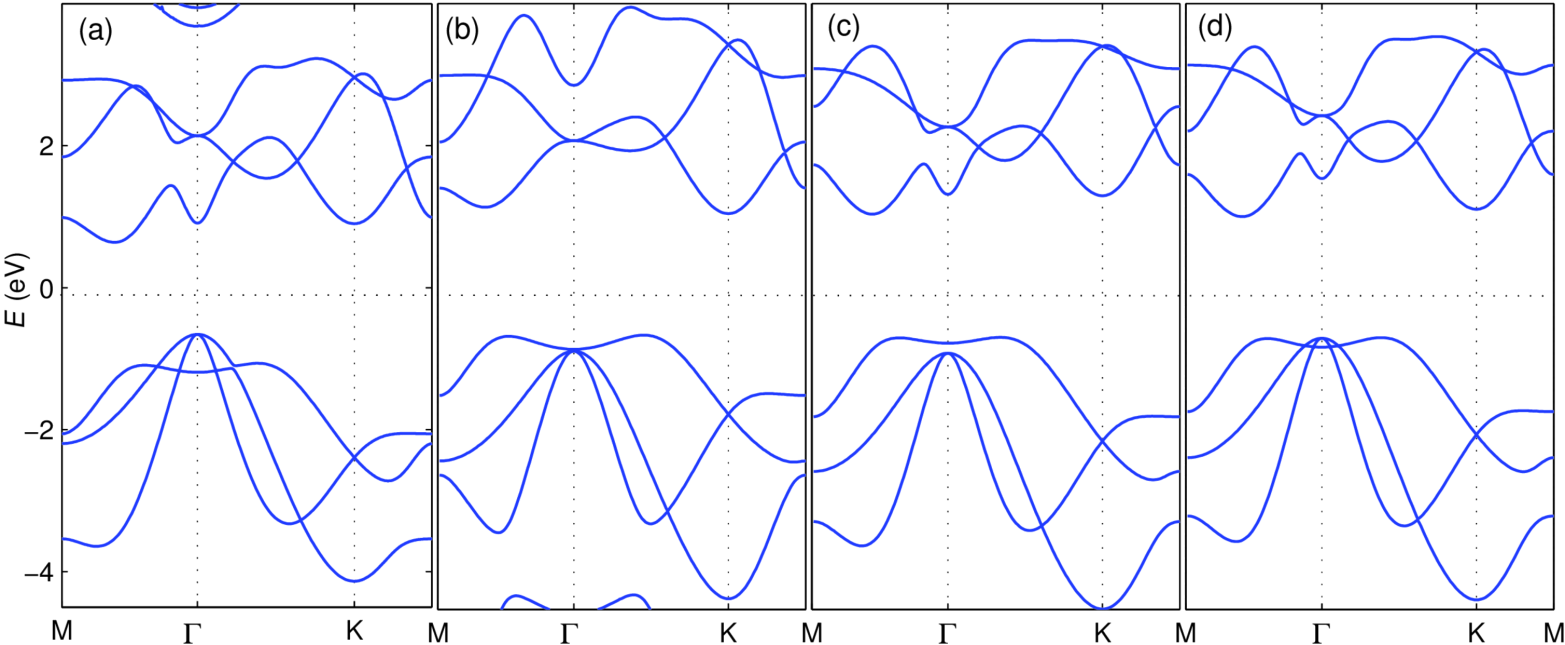
Comparison between different calculations of the band structure of monolayer antimonene as obtained from (a) VASP and CRYSTAL with different basis sets: (b) ANO, (c) WTBS, and (d) WTBS+ANO (see text for details). The lattice structure relaxed with VASP has been considered for all cases and SOC has not been included in the calculations.

Figure 1 presents the DFT band structure of a single layer of antimonene without SOC, in the framework of the Perdew–Burke–Ernzerhof local density approximation [42] to the functional for different basis sets. Panel (a) shows the results using the VASP [12] package. Calculations are performed with a plane-wave cutoff of 400 eV on a 15 × 15 × 1 Monkhorst–Pack *k*-point mesh. For structural relaxation, all atoms are allowed to relax until atomic forces are smaller than 0.01 eV/Å.

**Figure 2:**
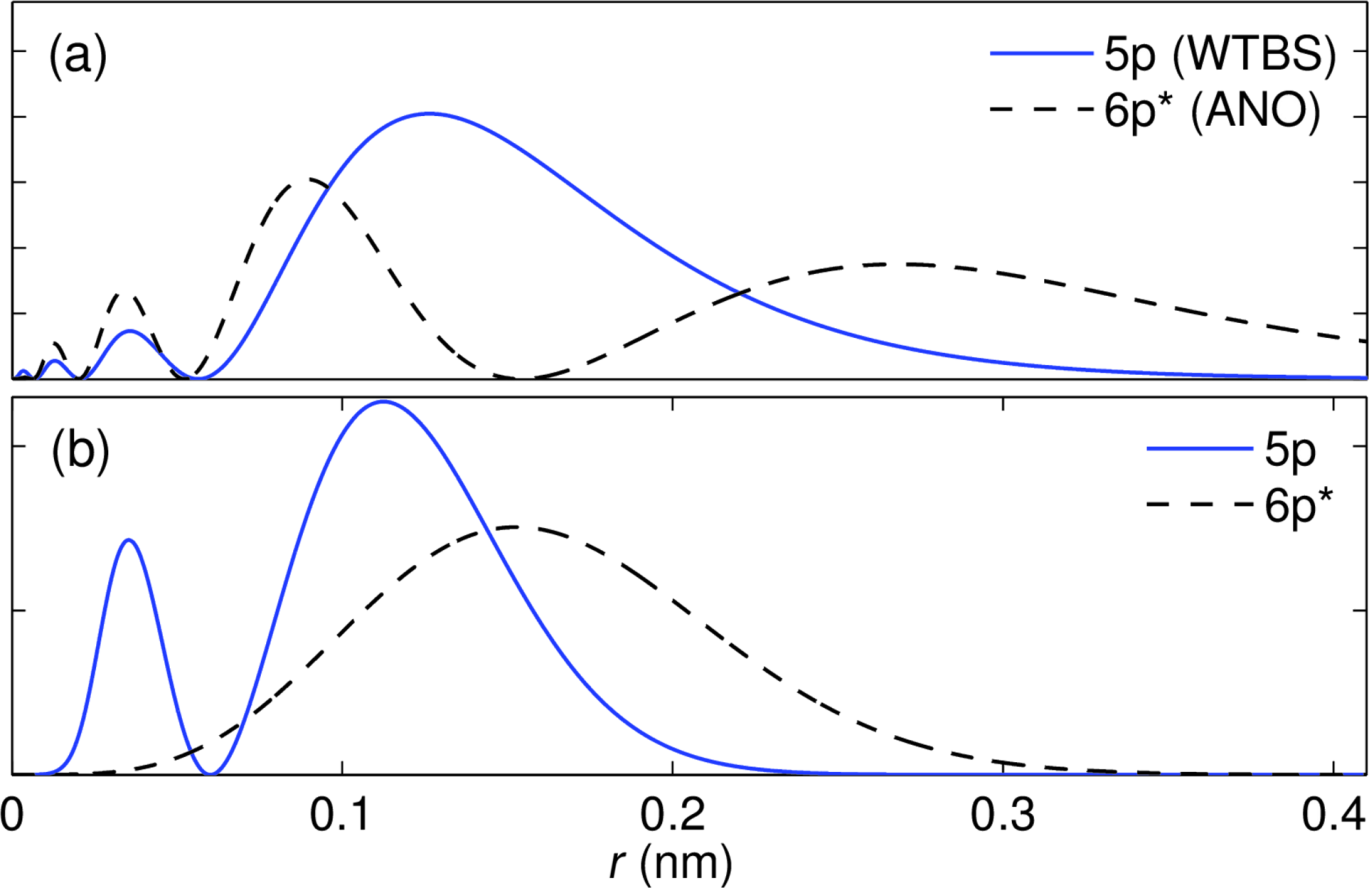
Radial probability density of two selected elements of the AE and small-core basis sets used in the calculations. (a) Solid blue curve corresponds to the 5p shell of the WTBS while the dashed black represents the 6p (virtual) shell in the ANO basis set (see text). (b) Same as in (a), the last partially occupied and the first empty (virtual) shell of the small-core pseudopontential basis set (see text), showing the lack of nodal structure required, in principle, for an appropriate SOC calculation.

In agreement with previous studies for free standing antimonene [43], we obtain an in plane lattice constant of the relaxed structure *a* = 4.12 Å and a buckling height *h* = 1.64 Å. Panels (b) and (c) show the band structure obtained with CRYSTAL using two standard AE basis sets properly converged in the number of elements. The former is based on relativistically contracted atomic natural orbitals [44–45] (ANO) and the latter belongs to the family of well-tempered basis sets [46] (WTBS). Examples of (the radial part of) basis elements from these two basis sets are shown in Figure 2a. For the sake of simplicity in the discussions and since no significant differences have been found, the same lattice structure (relaxed with VASP in presence of SOC) and same functional has been used in all band structure calculations. When compared to the VASP results, ANO bands turn out not too satisfactory at the high symmetry Γ point where the ordering of degenerate and non-degenerate bands is not reproduced. For other *k*-points across the Brillouin zone the results are comparatively better. The WTBS results shown in (c) manifest a significant improvement, particularly for the conduction bands, although the ordering of the valence bands is still not the correct one at the Γ point. Interestingly, we have found out that a combination of both ANO and WTBS basis sets [panel (d)] improves the band structure to the point of making it essentially similar to the VASP result. Here we have complemented the WTBS basis with additional valence orbitals from the ANO basis set. Adding this flexibility to the basis, even the flat valence band falls below the degenerate ones at the Γ point. This band structure corresponds to that of a semiconductor with an indirect gap, as previously reported [34]. The use of a hybrid functional such as HSE06 [37] will certainly increase the value of the gap, but we are not concerned with this issue here.

**Figure 3:**
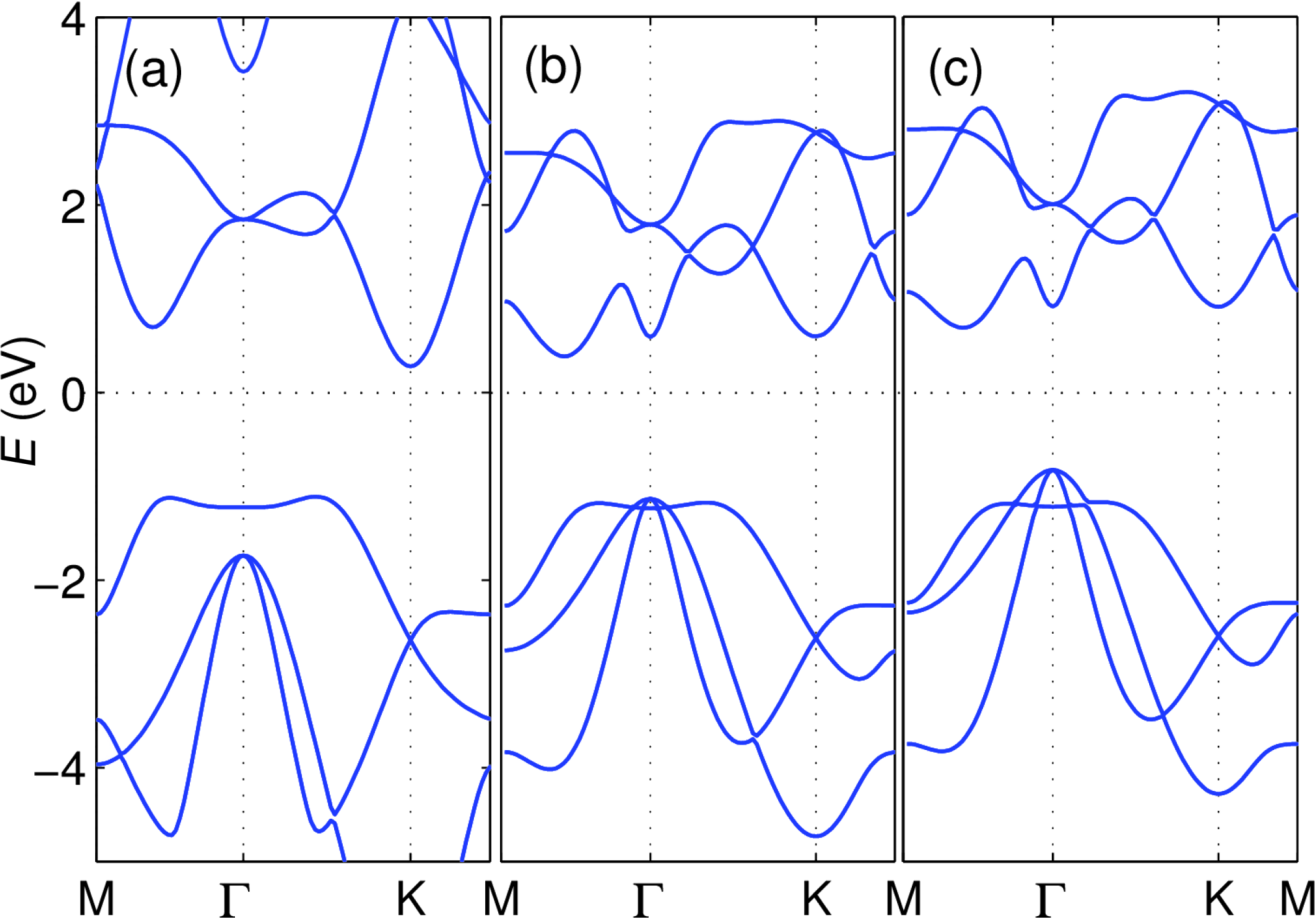
Comparison between different calculations of the band structure of monolayer antimonene as obtained from CRYSTAL with different pseudopotential basis sets: (a) large-core (46 electrons) and minimal basis set, (b) large- core as in (a) but a larger basis set, and (c) small-core and large basis set (see text for details). The lattice structure relaxed with VASP has been used for all cases and SOC was not included in the calculations.

Figure 3 shows the band structure obtained with CRYSTAL using three different pseudopotential basis sets. From (a) to (c) the quality of the basis set is improved. Starting from the bands obtained with a large effective core (46 electrons) and a minimal 4 element basis set [sp^3^] [47–48] [shown in panel (a)], we first increase the number of valence basis elements to 8 [2s2p^3^] [49] [see panel (b)], and then decrease the number of effective core electrons down to 28, while keeping a large 23 element basis set for the valence electrons [4s3p^3^2d^5^] [50] [panel (c)]. Figure 2b shows the radial part of the last two p-orbitals (or p-type CGTOs) in this third basis set. As can be observed, the nodal structure near the origin is absent. The shorter radial extension when compared to the corresponding orbital-like AE CGTOs (in particular for the one labeled 6p) is due to the fact that one cannot naively make a one-to-one correspondence between atomic orbitals and these basis elements. Except for the results using the minimal basis set, where the ordering of the bands is not the correct one (keeping in mind that the lattice parameters are the same for all calculations), the results of the other two calculations are fairly satisfactory. In particular, the small-core basis set bands in Figure 3c match nicely those obtained with VASP in Figure 1a. The slight discrepancies between the bands in Figure 1a and Figure 3c on one hand and the bands in Figure 1d on the other can be due to the use of pseudopotentials in the former two or to an inaccurate closure relation of the AE basis set in the latter. We will not address this issue any further here. Finally, we stress that our proposed implementation is not restricted to any specific Gaussian-type basis set. As an advantage when compared to, e.g., TB calculations, it can capture the SOC effect for more flexible and larger basis sets when a minimal basis does not give satisfactory results in a band structure calculation, as is the case shown in Figure 3a.

**Figure 4:**
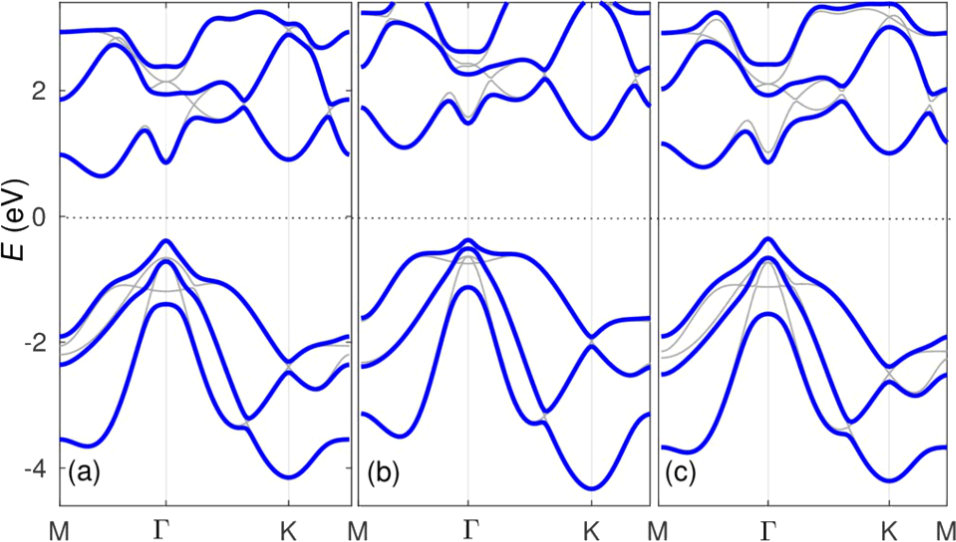
Comparison between different calculations of the band structure of monolayer antimonene including SOC: (a) VASP code, (b) AE basis set (WTBS+ANO), and (c) small-core pseudopotential basis set. As a reference, thin gray curves indicate the bands before adding SOC. The same lattice structure relaxed with VASP has been used for all cases.

Now that we have verified that we can obtain essentially the same band structure with two different DFT codes and three different basis sets (plane waves, AE, and pseudopotential ones), we add SOC. Figure 4 shows the band structure obtained with VASP [panel (a)] and with our proposed implementation, applied to the WTBS+ANO basis set [panel (b)] and to the small core pseudopotential basis set [panel (c)]. The AE basis set bands share all the features of the VASP bands, except a slightly larger gap which originates from the calculations without SOC. For the pseudopotential basis set, as discussed above, we have increased the effective nuclear potential by a factor of 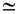
65 (for this specific basis set) that makes the bands look as similar as possible to those in panel (a). As can be seen, these last bands, tuned by a single parameter, are essentially indistinguishable from the VASP bands. As can be observed, the sizable SOC of Sb changes the previous band structure calculated without SOC considerably, removing degeneracies, but not in a qualitative manner. The changes are, however, not so trivial for few-layered antimonene as shown in the next section.

#### Multilayer antimonene

As an elemental bulk material, Sb appears to be a topological semi-metal due to an inversion of the ”natural” bulk band order [1]. Despite the absence of a bulk gap, its non-zero topological invariant guarantees that antimony features protected topological surface states (TSS), although coexisting with bulk bands at the Fermi energy [51–54]. Sb(111) in thin film form could become, in principle, a 3D (TI) if quantum confinement opened a gap in the bulk bands. However, for sufficiently thin films, the TSS situated on opposite surfaces can get coupled which degrades or even destroys the TSS exotic properties such as their expected protection against backscattering. Ultimately, a single Sb(111) layer or monolayer antimonene even becomes a trivial semiconductor, as discussed in the previous section. Previous calculations have shown that the decoupling of the TSS requires a minimum of 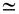
7 layers [54–55]. In between the semiconductor monolayer and the 7-layered antimonene a crossover occurs, where claims of the existence of a 2D topological insulator have also been reported, but we do not pursue the investigation of this issue here [55]. When TSS are decoupled and the gap at the Dirac point closes down, the Fermi energy crosses the Dirac cone above the Dirac point, but also crosses 6 surface state pockets and 3 bulk pockets (see, e.g., [51]).

**Figure 5:**
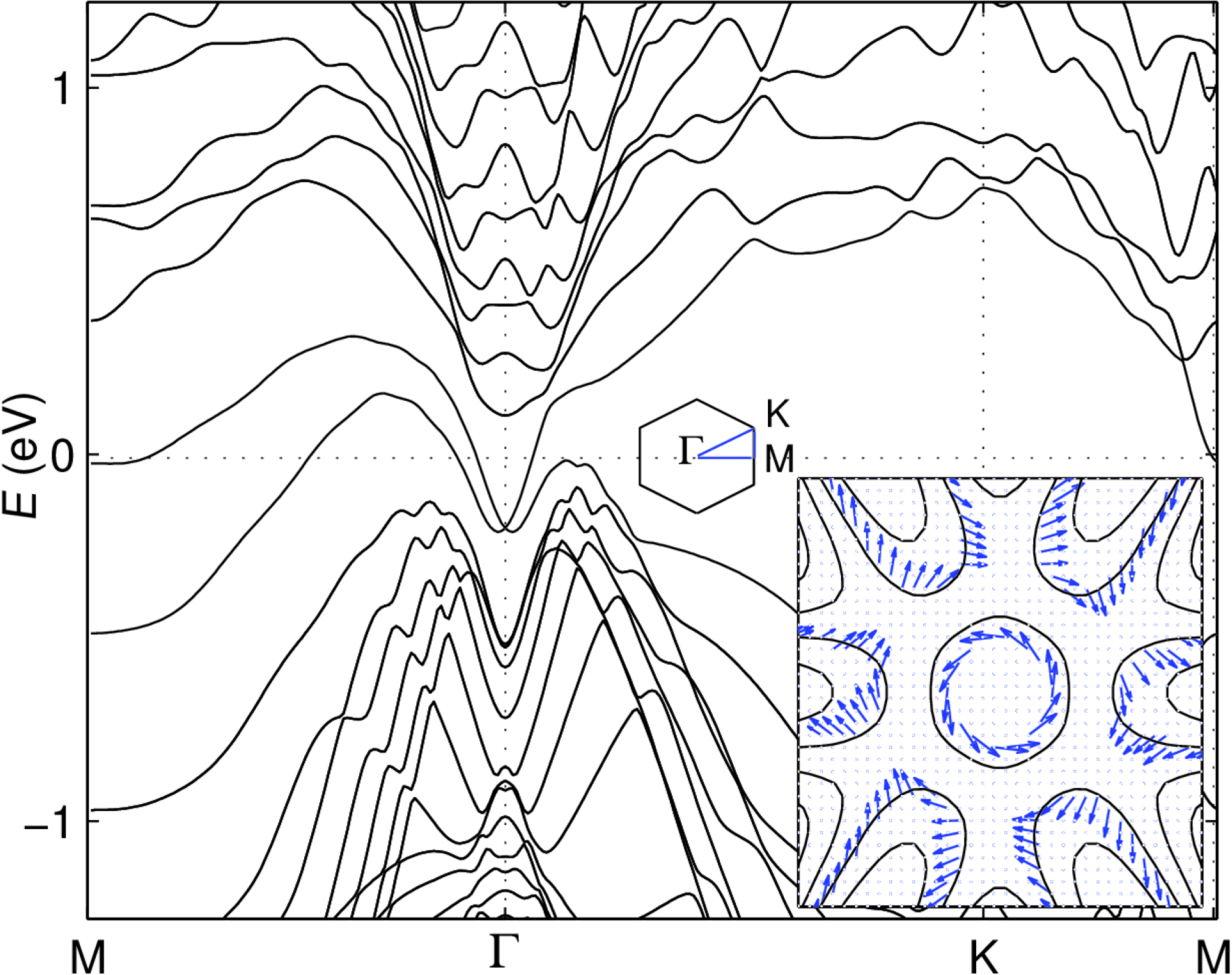
Band structure of 9 layers, ABC stacking Sb(111) films as obtained from small core pseudopotential basis. The spin texture around the Γ point is presented in the inset.

It has been shown that multilayer antimonene with hexagonal structure, prefers ABC stacking and is more stable than other allotropes for thicknesses larger than 3 layers [43]. In the relaxed structure of 9 layer antimonene, the lattice constant is *a* = 4.27 Å and the intra- and interlayer distances are *h* = 1.52 Å and *d* = 3.68 Å, respectively. In Figure 5 we show the band structure of 9 layers of antimonene including SOC, as obtained with the small-core pseudopotential basis set and the same enhancement factor as in the previous section. The results compare rather well down to any practical detail with those reported in the literature. In the inset of Figure 5 we show that the spin texture of the surface Dirac cone states around the Γ point and of the states in the nearby pockets, comes out as expected [51,56]. This provides further evidence that not only the band structure is reproduced at first glance, but also the wave functions are properly evaluated. This non-trivial example illustrates the practicality of our proposed single-parameter implementation, when AE basis sets are computationally demanding.

### Bi(111) monolayers

**Figure 6:**
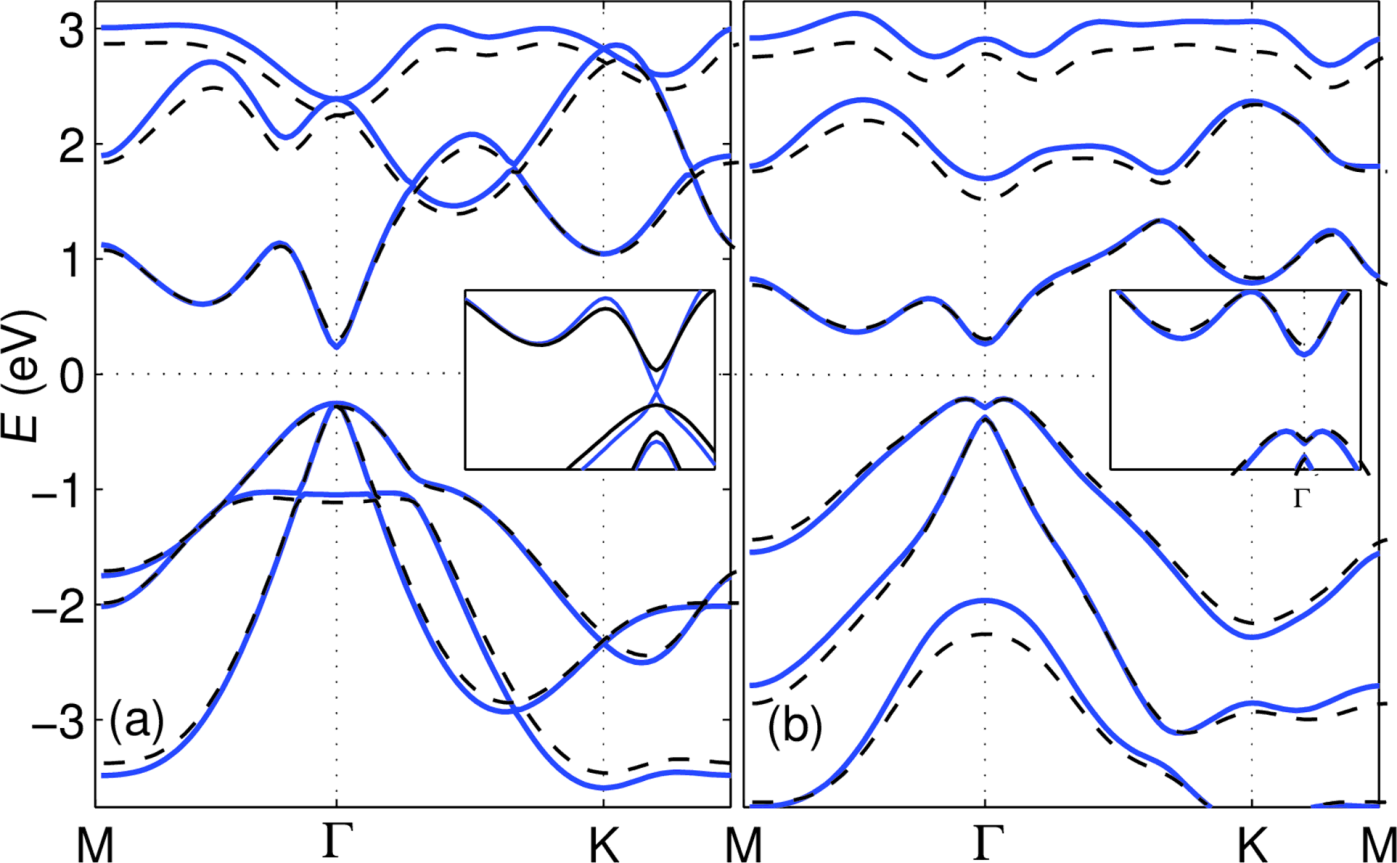
Band structure of a Bi(111) monolayer, obtained from VASP (dashed black) and small core pseudopotential basis set (solid blue) (a) without and (b) with SOC. Inset of panel (a) shows closing (blue) and reopening (black) of the gap with two parameter values of 70 and 100, respectively. Inset of panel (b), using the final parameter value of 120, is the same bands zoomed in near the Γ point showing the similarity of the inverted bands compared to the VASP result.

A monolayer of Bi(111) was one of the first 2D crystals predicted to be a 2D TI [27] and with actual chances to be experimentally isolated and characterized. However, only a few reports have confirmed the non-trivial topological character of this material [57–60]. Having seen the trivial bands of antimonene monolayer changing to nontrivial in multilayers, the band inversion of Bi(111) is addressed in this section. Using different DFT packages, a wide range of structural parameters have been reported for Bi(111). Being aware of the sensitivity of the band structure to the exact atomic structure and for the sake of comparison we use a lattice constant *a* = 4.33 Å and a buckling height *h* = 1.74 Å as reported in a similar VASP calculation [61]. Figure 6 shows the band structure of Bi(111) monolayer, as obtained from VASP (dashed black), and that calculated with a small-core pseudopotential basis set (solid blue), as obtained with our implementation. Starting from very similar band structures without SOC (a), the proposed implementation of SOC gives a band structure in close resemblance with the one obtained from VASP (b) (the multiplicative factor needed to increase the nuclear potential is 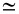
120 in case of this specific basis set). Increasing the multiplicative factor of our implementation from zero to two intermediate values (for example 70 and 100), as shown in the inset of Figure 6a, one can follow the evolution of the band structure from trivial to nontrivial bands and the ”Mexican hat” shaped valence band in Figure 6b. The band inversion at the Γ point is evident. However, this visual evidence is not sufficient to prove that this system is a topological insulator and a calculation of the Z2 number demonstrates that this is case.

Regardless of the shortcomings of tight binding method which led us towards this implementation of SOC, here, we want to compare the order of magnitude of tight binding SOC parameters with our SOC correction. In TB implementation, only one multiplicative parameter serves as the radial correction of SOC and this factor is much smaller than our multiplicative factors. The TB parameter entirely replaces the actual evaluation of the radial integral in Equation 5. However, our multiplicative factor is used to correct the radial integral which we actually perform for all matrix elements. The large numbers that we report come about because the radial matrix elements can be very small due to the lack of nodal structure of the basis elements, but, in the end, the correction parameter that accompanies the angular part for the valence orbitals will be in the same order of magnitude as what is reported for similar tight binding models.

## Conclusion

We have presented an implementation of SOC suitable for DFT band structure calculations based on CGTOs basis sets. We evaluate both angular and radial part of the SOC relativistic correction to the Hamiltonian, considering the spherical harmonics and CGTOs as the angular and radial part of the basis functions, respectively. The evaluated SOC term is then added after a standard non-relativistic (or scalar relativistic) self-consistent calculation. We have shown that if the AE band structure is in good agreement with plane-wave bands without SOC, when our implementation is applied, it can reproduce the band structure obtained from the VASP code (used as a reference) to our satisfaction. Although we have only tested it in the cases of antimonene and Bi(111), we see no reason why it should not work for other elemental and compound materials, since it is essentially first-principles and SOC is an intra-atomic correction. We have also shown that a simple modification (by a multiplicative factor) of the effective nuclear potential makes this implementation applicable for pseudopotential basis sets which lack nodal structure. Remarkably, the results obtained in this last manner fit even better those obtained with plane-waves and the VASP code. In contrast to standard TB implementations where the SOC parameter acts on the valence orbitals of a minimal basis set, our method does not consider any pre-assumptions for the basis elements. Note that using GTOs as basis elements, the so-called valence orbital might be split into two or more basis elements to improve the quality of the band structure. Our proposed approach is a practical way of including SOC to standard DFT non-relativistic band structure calculations based on localized basis sets.
